# Corrigendum

**DOI:** 10.1111/nph.18843

**Published:** 2023-04-01

**Authors:** Wubei Zong, Ding Ren, Minghui Huang, Kangli Sun, Jinglei Feng, Jing Zhao, Dongdong Xiao, Wenhao Xie, Shiqi Liu, Han Zhang, Rong Qiu, Wenjing Tang, Ruqi Yang, Hongyi Chen, Xianrong Xie, Letian Chen, Yao-Guang Liu, Jingxin Guo

**Keywords:** flowering time, heading date, photoperiod sensitivity, rice, short‐day plant

*New Phytologist* **229** (2021), 1635–1649, doi: 10.1111/nph.16946.

Since its publication, errors have been identified in the figures of Zong *et al*. (2021).

In Fig. 2(a) and Supporting Information Fig. S8(b) images of composite figures were misplaced during assembly. The authors present their original photographs, in corrected figures below. The description of data and conclusions in the paper remain unchanged. Furthermore, the Y1H conclusions have been validated by further independent research (Su *et al*., 2022).

To present the data in Figs 1(b) and S9(a) more clearly, all yeast two‐hybrid assays have been redone by the authors, and these are consistent with the original work. All data are shown in three concentration gradients (10^0^×, 10^−1^×, 10^−2^×), which were grown on the same plate (corrected Figs 1b, S9a below).

We sincerely apologize to our readers for these mistakes.

**Corrected Figs 1 and 2 and Supporting Information Figs S8 and S9**

**Fig. 1:**
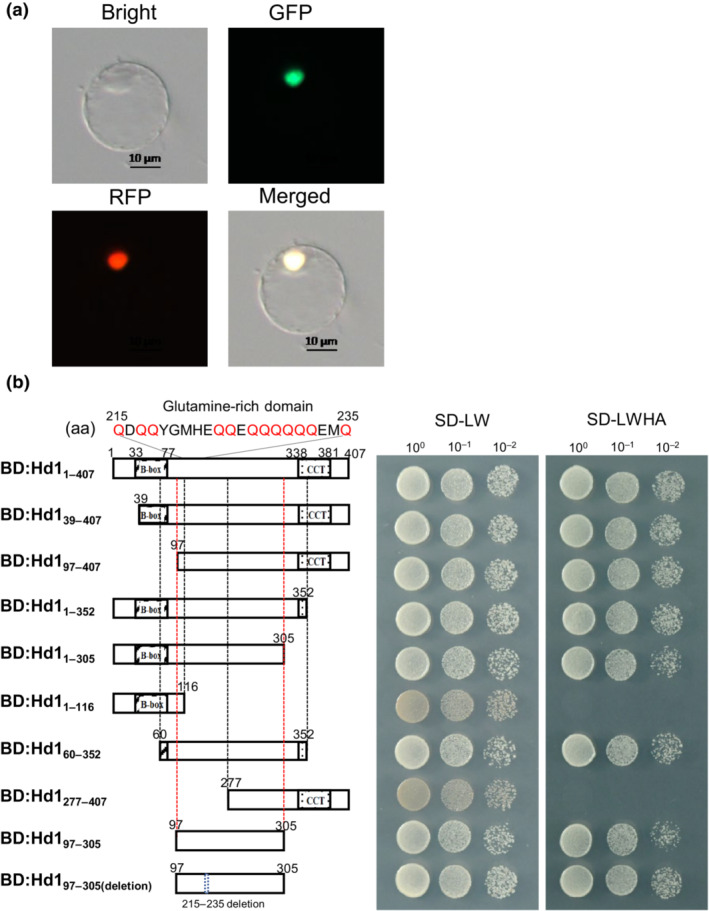
Characterization of Hd1 protein. (a) Subcellular localization of Hd1‐eGFP fusion protein in rice protoplasts. The TDR‐RFP fusion protein served as the nuclear marker. Bar, 10 μm. (b) Transcriptional activation assays of Hd1 and its truncated derivatives in the yeast GAL4 system. BD is the GAL4‐DNA‐binding domain. The red Q indicates glutamine (Gln). SD‐LW, SD‐Leu‐Trp, SD‐LWHA, SD‐Leu‐Trp‐His‐Ade. 10^0^×, 10^−1^×, 10^−2^× diluted cells were spotted onto synthetic defined SD‐LW (left) and SD‐LWHA (right) medium.

**Fig. 2:**
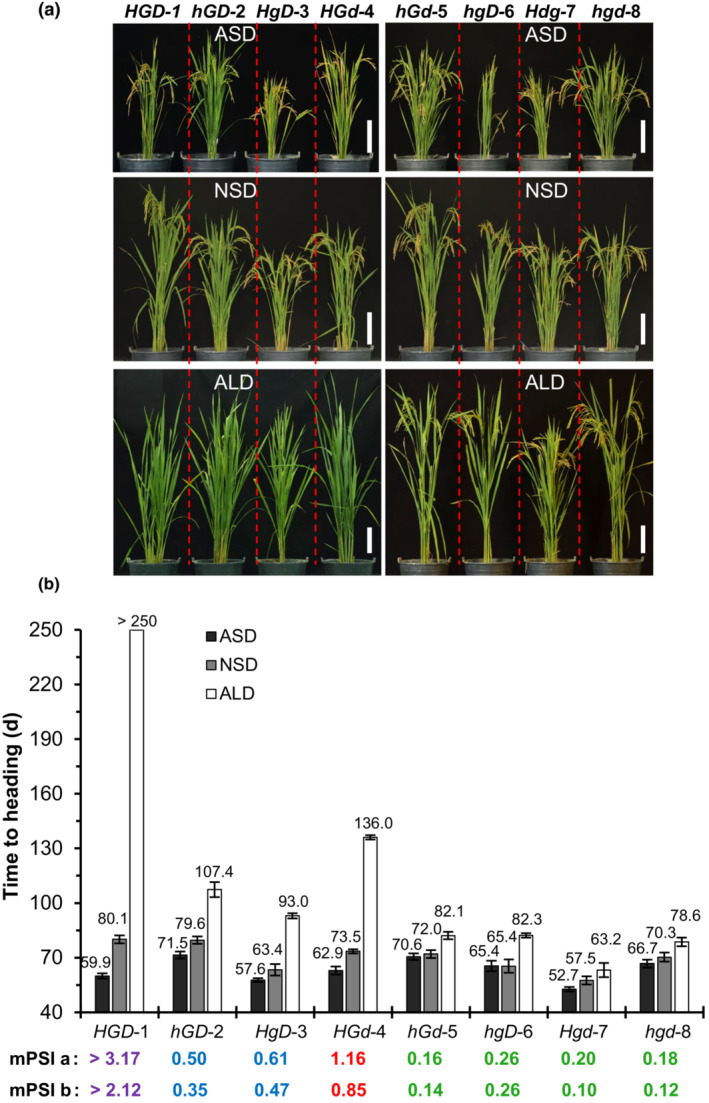
Eight isogenic lines with combinations of the *Hd1*, *Ghd7* and *DTH8* alleles generated by gene knockout and crossing. (a) Phenotypes of the lines (97‐d‐old plants) under artificial short‐day (ASD), natural short‐day (NSD) and artificial long‐day (ALD) conditions in Guangzhou. Bar, 25 cm. In *NHLD* background (*HGD*‐1), which has strong photoperiod sensitivity (PS), *Hd1*, *Ghd7* and *DTH8* were knocked out, respectively, via CRISPR/Cas9 to generate mutant lines (*hGD*‐2, *HgD*‐3, *HGd*‐4). Then a set of eight isogenic lines with all combinations of *Hd1*, *Ghd7* and *DTH8* alleles were developed by crossing among *hGD*‐2, *HgD*‐3 and *HGd*‐4 to generate *hGd‐5*, *hgD‐6*, *Hgd‐7* and *hgd‐8*. (b) Heading dates of the lines in the different day‐length conditions, and their modified PS index (mPSI) values based on ASD (a) or NSD conditions (b). Data are means SD (*n* = 50). The degrees of PS are classified into very strong (purple), strong (red), moderate (blue) and weak (green) based on the mPSI values.

**Fig. S8:**
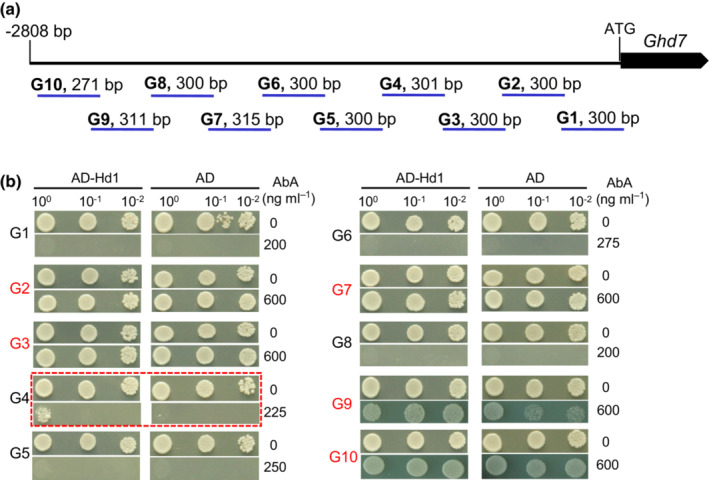
Test of the interaction of Hd1 with the *Ghd7*‐promoter region. (a) Fragments of the *Ghd7*‐promoter region used to test the interaction with Hd1 using yeast one‐hybrid assays. (b) Results of the yeast one‐hybrid assays. Yeast cells were applied to the media in three dilutions. Fragments shown in red had self‐activation activity. G4 (red dashed line) showed a weak interaction with Hd1.

**Fig. S9:**
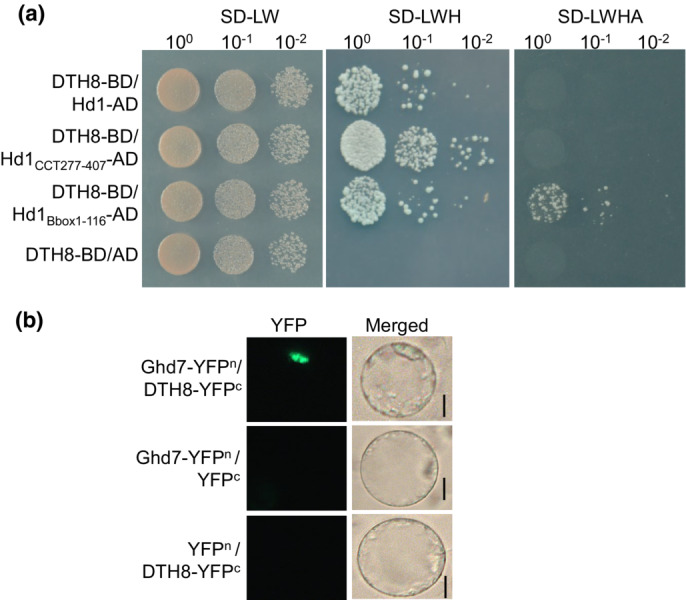
Verification of the interactions among Hd1, DTH8 and Ghd7. (a) AD‐Hd1 interacted with BD‐DTH8 in yeast two‐hybrid assays. The empty vector expressing the AD domain alone was the negative control. SD‐LW, SD‐Leu‐Trp, SD‐LWH, SD‐Leu‐Trp‐His, SD‐LWHA, SD‐Leu‐Trp‐His‐Ade. 10^0^×, 10^−1^×, 10^−2^× diluted cells were spotted onto synthetic defined SD‐LW (left) and SD‐LWH (middle) and SD‐LWHA (right) medium. (b) BiFC assays for the interaction of Ghd7 and DTH8 in rice protoplasts. Bar, 20 μm.

## References

[nph18843-bib-0001] Su Q , Zhang F , Xiao Y , Zhang P , Xing H , Chen F . 2022. An efficient screening system to identify protein‐protein or protein‐DNA interaction partners of rice transcription factors. Journal of Genetics and Genomics 49: 979–981. 35218975 10.1016/j.jgg.2022.02.007

[nph18843-bib-0002] Zong W , Ren D , Huang M , Sun K , Feng J , Zhao J , Xiao D , Xie W , Liu S , Zhang H *et al*. 2021. Strong photoperiod sensitivity is controlled by cooperation and competition among Hd1, Ghd7 and DTH8 in rice heading. New Phytologist 229: 1635–1649. 33089895 10.1111/nph.16946 PMC7821112

